# Association between CYP1A2 and CYP1B1 Polymorphisms and Colorectal Cancer Risk: A Meta-Analysis

**DOI:** 10.1371/journal.pone.0100487

**Published:** 2014-08-12

**Authors:** Xiao-Feng He, Jie Wei, Zhi-Zhong Liu, Jian-Jun Xie, Wei Wang, Ya-Ping Du, Yu Chen, Hui-Qiang Si, Qing Liu, Li-Xia Wu, Wu Wei

**Affiliations:** 1 Department of Research, Peace Hospital of Changzhi Medical College, Changzhi, Shanxi Province, China; 2 Department of Clinical laboratory, Sun Yat-Sen Memorial Hospital, Sun Yat-Sen University, Guangzhou, Guangdong Province, China; 3 Department of Gastroenterology, The Second People's Hospital of Zhuhai, Zhuhai, Guangdong Province, China; 4 Department of Hematology, Peace Hospital of Changzhi Medical College, Changzhi, Shanxi Province, China; University of Michigan, United States of America

## Abstract

**Background:**

The previous published data on the association between CYP1A2*F (rs762551), CYP1B1 Leu432Val (rs1056836), Asn453Ser (rs180040), and Arg48Gly (rs10012) polymorphisms and colorectal cancer risk remained controversial.

**Methodology/Principal Findings:**

The purpose of this study is to evaluate the role of CYP1A2*F, CYP1B1 Leu432Val, Asn453Ser, and Arg48Gly genotypes in colorectal cancer susceptibility. We performed a meta-analysis on all the eligible studies that provided 5,817 cases and 6,544 controls for CYP1A2*F (from 13 studies), 9219 cases and 10406 controls for CYP1B1 Leu432Val (from 12 studies), 6840 cases and 7761 controls for CYP1B1 Asn453Ser (from 8 studies), and 4302 cases and 4791 controls for CYP1B1Arg48Gly (from 6 studies). Overall, no significant association was found between CYP1A2*F, CYP1B1 Leu432Val, Asn453Ser, and Arg48Gly and colorectal cancer risk when all the eligible studies were pooled into the meta-analysis. And in the subgroup by ethnicity and source of controls, no evidence of significant association was observed in any subgroup analysis.

**Conclusions/Significance:**

In summary, this meta-analysis indicates that CYP1A2*F, CYP1B1 Leu432Val, Asn453Ser, and Arg48Gly polymorphisms do not support an association with colorectal cancer, and further studies are needed to investigate the association. In addition, our work also points out the importance of new studies for CYP1A2*F polymorphism in Asians, because high heterogeneity was found (dominant model: *I* ^2^ = 81.3%; heterozygote model: *I* ^2^ = 79.0).

## Introduction

Sporadic colorectal cancer (CRC) is considered to be a multifactorial disease, in which multiple exposures to endogenous factors and dietary carcinogens interact with individual genetic background in a complex manner resulting in modulation of the risk [1]. In 2010, an estimated 142,570 new cases will be diagnosed and 51,370 deaths will occur in the whole world [2]. Epidemiologic studies on Western populations have emphasized the large contribution of food and lifestyle to sporadic CRC risk [3]–[7]. High-fat and low-fiber diets, as well as alcohol, tobacco, and red or processed meat consumption, have been shown to produce high levels of polycyclic aromatic hydrocarbons and heterocyclic aromatic amines. These procarcinogenic agents are potentially very harmful and may play a key role in the malignant transformation of cells by interacting with DNA [8]. It has been proposed that this risk may be due to carcinogenic polycyclic aromatic hydrocarbons (PAHs) and heterocyclic amines produced when meat is cooked at high temperatures [9].

CYP1B1 gene is located on chr2p22-p21, which is involved in the metabolic activation of polycyclic aromatic hydrocarbons (PAHs) including benzo(a)pyrene and dimethylbenz(a)anthracene (DMBA), but with a product distribution that is distinct from CYP1A1 [10], [11]. Several lines of evidence suggest that CYP1B1 plays a role in carcinogenesis. CYP1B1 is commonly over-expressed inhumanmalignancies [12] and activates a variety of carcinogens. For example, CYP1B1 catalyzes both the formation of dihydrodiols of specific PAHs and their subsequent oxidation to carcinogenic dihydrodiol epoxides [13]. In humans, CYP1B1 is genetically polymorphic and more than 50 single nucleotide polymorphisms (SNPs) have been reported so far, of which certain deleterious mutations are associated with primary congenital glaucoma [14]. Of the most common SNPs of CYP1B1 gene, four have been reported to result in amino acid substitutions including Arg by Gly at codon 48 (rs10012), Leu by Val at codon 432 (rs1056836) and Asn by Ser at codon 453 (rs1800440). CYP 1A2 is an important gene in catalyzing 2- and 4-hydroxylations of estrogens [40]–[42] and metabolism of carcinogens [43]–[45]. CYP1A2*1C, located in the 5′-non-coding promoter region of CYP1A2, was reported to be associated with decreased enzyme inducibility in Japanese smokers but seems to be very rare [46].

To date, a number of molecular epidemiological studies have been done to evaluate the association between CYP1A2*F, CYP1B1 Leu432Val, Asn453Ser, and Arg48Gly polymorphisms and colorectal cancer risk in diverse populations [15]–[29], [31], [32], [34]–[39]. However, the results were inconsistent or even contradictory. Therefore, we performed a comprehensive meta-analysis by including the most recent and relevant articles to identify statistical evidence of the association between CYP1A2*F, CYP1B1 Leu432Val, Asn453Ser, and Arg48Gly polymorphisms and risk of colorectal cancer that have been investigated. Meta-analysis is a powerful tool for summarizing the different studies. It can not only overcome the problem of small size and inadequate statistical power of genetic studies of complex traits, but also provide more reliable results than a single case–control study.

## Materials and Methods

### Identification and eligibility of relevant studies

A comprehensive literature search was performed using the PubMed, CNKI, and Medline database for relevant articles published (the last search update was Sep 10, 2013) with the following key words “CYP1A2”, “CYP1B1”, “polymorphism”, “Variant”, or “Mutation”, and “Colorectal”. In addition, studies were identified by a manual search of the reference lists of reviews and retrieved studies. We included all the case–control studies and cohort studies that investigated the association between CYP1A2*F, CYP1B1 Leu432Val, Asn453Ser, and Arg48Gly polymorphisms and colorectal cancer risk with genotyping data. All eligible studies were retrieved, and their bibliographies were checked for other relevant publications.

### Inclusion criteria

The included studies have to meet the following criteria: (1) only the case–control studies or cohort studies were considered; (2) evaluated the CYP1A2*F, CYP1B1 Leu432Val, Asn453Ser, and Arg48Gly polymorphisms and the risk of colorectal cancer; (3) the genotype distribution of the polymorphism in cases and controls were described in details and the results were expressed as odds ratio (OR) and corresponding 95% confidence interval (95% CI). Major reasons for exclusion of studies were as follows: (1) not for cancer research; (2) only case population; (3) duplicate of previous publication (When the same patient population was used in several publications, only the most recent, largest or complete study was included following careful examination).

### Data extraction

Information was carefully extracted from all eligible studies independently by two investigators according to the inclusion criteria listed above. The following data were collected from each study: first author's name, year of publication, country of origin, ethnicity, source of controls (population-based controls, hospital-based controls, and family-based controls), and numbers of cases and controls in the CYP1A2*F, CYP1B1 Leu432Val, Asn453Ser, and Arg48Gly genotypes whenever possible. Ethnicity was categorized as “Caucasian” and “Asian”. When one study did not state which ethnic groups was included or if it was impossible to separate participants according to phenotype, the sample was termed as “mixed population”. We did not define any minimum number of patients to include in this meta-analysis. Articles that reported different ethnic groups and different countries or locations, we considered them different study samples for each category cited above.

### Statistical analysis

Crude odds ratios (ORs) together with their corresponding 95% confidence intervals (95% CIs) were used to assess the strength of association between the CYP1A2*F, CYP1B1 Leu432Val, Asn453Ser, and Arg48Gly polymorphisms and colorectal cancer risk. The pooled ORs were performed for dominant model (CYP1A2*F: CY + YY vs. CC; CYP1B1 Leu432Val: Leu/Val + Val/Val vs. Leu/Leu; CYP1B1 Asn453Ser: Asn/Ser + Ser/Ser vs. Asn/Asn; CYP1B1 Arg48Gly: Arg/Gly + Gly/Gly vs. Arg/Arg), recessive model (CYP1A2*F: YY vs. CC + CY; CYP1B1 Leu432Val: Val/Val vs. Leu/Leu + Leu/Val; CYP1B1 Asn453Ser: Ser/Ser vs. Asn/Asn + Asn/Ser; CYP1B1 Arg48Gly: Gly/Gly vs. Arg/Arg + Arg/Gly), co-dominant model (CYP1A2*F: YY vs. CC and CY vs. CC; CYP1B1 Leu432Val: Val/Val vs. Leu/Leu and Leu/Val vs. Leu/Leu; CYP1B1 Asn453Ser: Ser/Ser vs. Asn/Asn and Asn/Ser vs. Asn/Asn; CYP1B1 Arg48Gly: Gly/Gly vs. Arg/Arg and Arg/Gly vs. Arg/Arg), and additive model (CYP1A2*F: Y vs. C; CYP1B1 Asn453Ser: Ser/Asn; CYP1B1 Asn453Ser: Ser vs. Asn; CYP1B1 Arg48Gly: Gly vs. Arg), respectively. Between-study heterogeneity was assessed by calculating *Q*-statistic (Heterogeneity was considered statistically significant if *P*<0.10) [47] and quantified using the *I* ^2^ value, Venice criteria [48] for the *I* ^2^ test included: “*I* ^2^<25% represents no heterogeneity, *I* ^2^ = 25–50% represents moderate heterogeneity, *I* ^2^ = 50–75% represents large heterogeneity, and *I* ^2^>75% represents extreme heterogeneity”. If results were not heterogeneous, the pooled ORs were calculated by the fixed-effect model (we used the *Q*-statistic, which represents the magnitude of heterogeneity between-studies) [49]. Otherwise, a random effect model was used (when the heterogeneity between-studies were significant) [50]. We also performed subgroup analysis by ethnicity and source of controls were conducted. Moreover, sensitivity analysis was performed by excluding a single study each time. We also ranked studies according to sample size, and then repeated this meta-analysis. Sample size was classified according to a minimum of 200 participants and those with fewer than 200 participants. The cite criteria were previously described [51]. HWE was calculated by using the goodness-of-fit test, and deviation was considered when *P*<0.05. Begg's funnel plots [52] and Egger's linear regression test [53] were used to assess publication bias. We opted for using ethnicity, source of controls, menopausal status, and sample size as possible different sources of heterogeneity. All of the calculations were performed using STATA version 10.0 (STATA Corporation, College Station, TX).

## Results

### Literature search and meta-analysis databases

**Figure 1:**
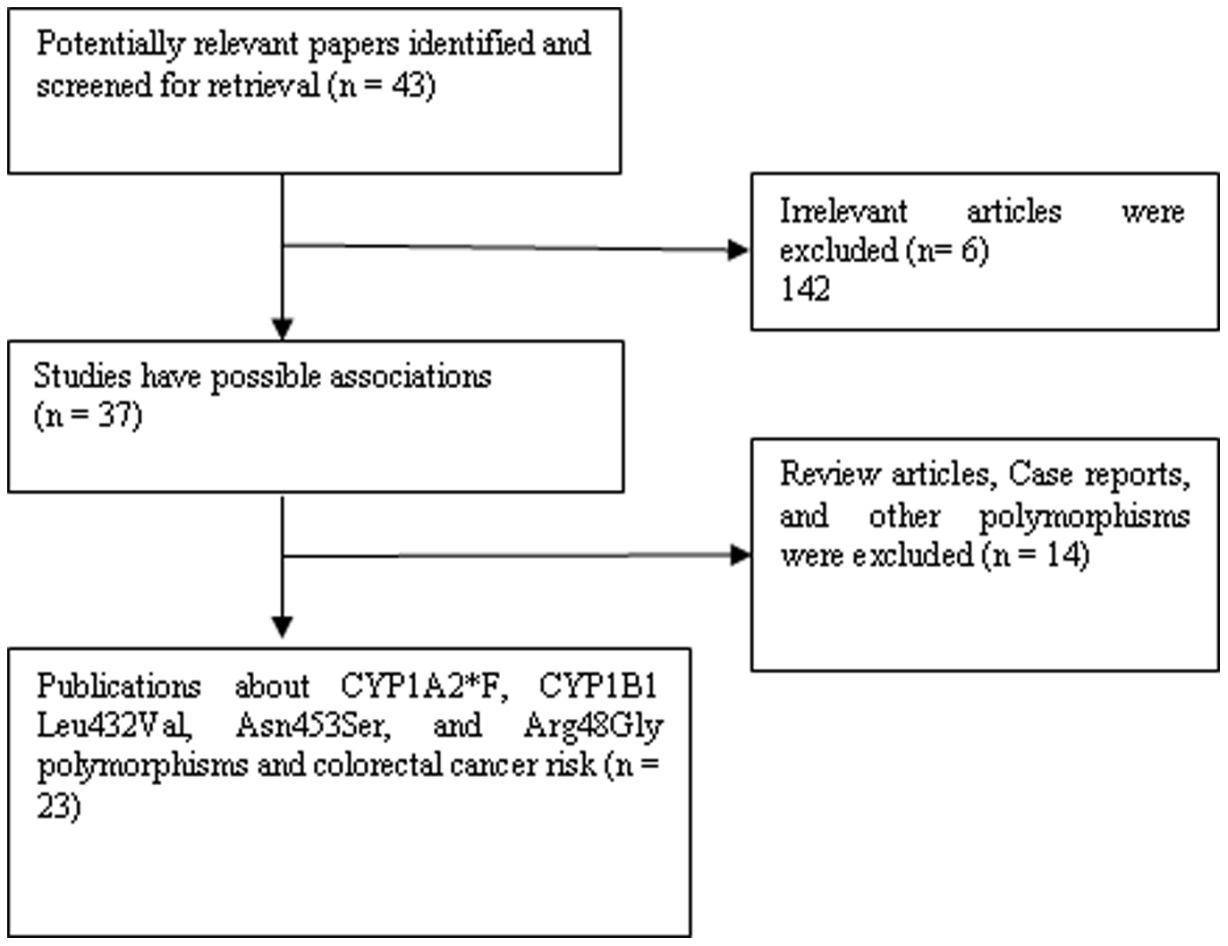
Study flow chart explaining the selection of the 23 eligible articles included in the meta-analysis.

Relevant publications were retrieved and preliminarily screened. As shown in **Fig. 1**, 43 publications were identified, among which 6 irrelevant papers were excluded. Thus, 37 publications were eligible. Among these publications, 14 articles were excluded because they were review articles, case reports, and other polymorphisms of CYP1A2 and CYP1B1. As summarized in **Table 1**, 23 articles with 39 studies were selected in this meta-analysis, including 5,817 cases and 6,544 controls for CYP1A2*F (from 13 studies), 9,219 cases and 10,406 controls for CYP1B1 Leu432Val (from 12 studies), 6,840 cases and 7,761 controls for CYP1B1 Asn453Ser (from 8 studies), and 4,302 cases and 4,791 controls for CYP1B1 Arg48Gly (from 6 studies). Among these studies, eight were Caucasians, four were Asians, and 1 mixed populations for CYP1A2*F. All studies were Caucasians except for one study was mixed population for CYP1B1 polymorphisms. The distribution of genotypes in the controls was consistent with Hardy–Weinberg equilibrium in all studies. All of the cases were pathologically confirmed.

**Table 1:** Main characteristics of all studies included in the meta-analysis.

First author/Year	Country	Ethnicity	SC	Genotype distribution						HWE	No. of case/control
				Cases			Controls				
				CC	CY	YY	CC	CY	YY		
CYP1A2*F											
Wang [15] 2012	USA	Mixed	FB	164	117	24	184	144	29	Y	305/357
Rudolph [16] 2011	German	Caucasian	PB	354	261	63	353	280	47	Y	678/680
Sainz [17] 2011	German	Caucasian	PB	872	735	157	887	732	167	Y	1764/1786
Cleary [18] 2010	Canada	Caucasian	PB	598	461	106	648	517	125	Y	1165/1290
Kobayashi [19] 2009	Japan	Asian	HB	53	40	11	96	94	35	Y	104/225
Saebø [20] 2008	Norway	Caucasian	HB	97	87	14	122	84	16	Y	198/222
Sachse [21] 2002	UK	Caucasian	PB	264	193	33	325	233	35	Y	490/593
Yoshida [22] 2007	Japan	Asian	HB	26	32	6	42	52	17	Y	64/111
Kiss [23] 2007	Hungary	Caucasian	HB	219	212	69	228	207	65	Y	500/500
Küry [24] 2007	France	Caucasian	HB	514	420	79	553	480	85	Y	1013/1118
Bae [25] 2006	Korea	Asian	HB	24	71	16	44	37	12	Y	111/93
Chen [26] 2005	China	Asian	PB	19	62	57	47	133	160	Y	138/340
Landi [27] 2005	Spain	Caucasian	HB	141	172	48	158	137	26	Y	361/321
CYP1B1 Leu432Val (rs1056836)											

First author/Year	Country	Ethnicity	SC	Genotype distribution						HWE	No. of case/control
				Cases			Controls				
				Leu/Leu	Leu/Val	Val/Val	Leu/Leu	Leu/Val	Val/Val		
Wang [39] 2012	USA	Mixed	FB	86	139	75	118	151	81	Y	300/350
Rudolph et al. [28] 2011	German	Caucasian	PB	220	320	128	224	339	106	Y	668/669
Sainz et al. [29] 2011	German	Caucasian	PB	237	339	143	245	358	110	Y	719/713
Cleary et al. [18] 2010	Canada	Caucasian	PB	391	547	224	424	617	250	Y	1162/1291
Hlavata et al. [31] 2010	Czech	Caucasian	HB	174	237	84	155	262	78	Y	495/495
Trubicka et al. [32] 2010	Poland	Caucasian	PB	214	275	108	206	265	127	Y	597/598
Sachse et al. [21] 2002	UK	Caucasian	PB	141	258	91	187	283	123	Y	490/593
Cotterchio et al. [34] 2008	Canada	Caucasian	PB	283	382	166	407	604	237	Y	831/1248
Küry et al. [35] 2007	France	Caucasian	PB	317	507	189	368	576	174	Y	1013/1118
Bethke et al. [36] 2007	UK	Caucasian	HB	519	1277	763	538	1365	792	Y	2559/2695
Huber [37] 2005	Australia	Caucasian	HB	14	28		112	225		Y	42/337
Landi [38] 2005	Spain	Caucasian	HB	128	151	64	101	139	59	Y	343/299
Asn453Ser (rs1800440)											

First author/Year	Country	Ethnicity	SC	Genotype distribution						HWE	No. of case/control
				Cases			Controls				
				Asn/Asn	Asn/Ser	Ser/Ser	Asn/Asn	Asn/Ser	Ser/Ser		
Rudolph [28] 2011	German	Caucasian	PB	467	187	22	452	202	26	Y	676/680
Sainz [29] 2011	German	Caucasian	PB	505	203	23	473	222	27	Y	731/722
Cleary [18] 2010	Canada	Caucasian	PB	775	354	34	897	349	46	Y	1163/1292
Hlavata [31] 2010	Czech	Caucasian	HB	353	134	8	320	163	12	Y	495/495
Cotterchio [34] 2008	Canada	Caucasian	PB	549	262	21	867	340	42	Y	832/1249
Bethke [36] 2007	UK	Caucasian	HB	1734	739	86	1,790	828	76	Y	2559/2694
Huber [37] 2005	Australia	Caucasian	HB	26	16		219	113		Y	42/332
Landi [38] 2005	Spain	Caucasian	HB	219	107	16	190	90	17	Y	342/297
Arg48Gly (rs10012)											

First author/Year	Country	Ethnicity	SC	Genotype distribution						HWE	No. of case/control
				Cases			Controls				
				Arg/Arg	Arg/Gly	Gly/Gly	Arg/Arg	Arg/Gly	Gly/Gly		
Rudolph [28] 2011	German	Caucasian	PB	329	295	55	322	299	54	Y	679/675
Sainz [29] 2011	German	Caucasian	PB	354	318	60	342	321	57	Y	732/720
Cleary [18] 2010	Canada	Caucasian	PB	593	478	92	651	529	112	Y	1163/1292
Trubicka [32] 2010	Poland	Caucasian	PB	261	266	70	265	265	67	Y	597/597
Cotterchio [34] 2008	Canada	Caucasian	PB	424	347	61	624	518	107	Y	832/1249
Landi [38] 2005	Spain	Caucasian	HB	169	101	29	161	87	10	Y	299/258

PB population-based studies, HB hospital-based studies, FB family-based studies, Y yes, N no, SC source of control, HWE Hardy–Weinberg equilibrium.

### Meta-analysis results

**Figure 2:**
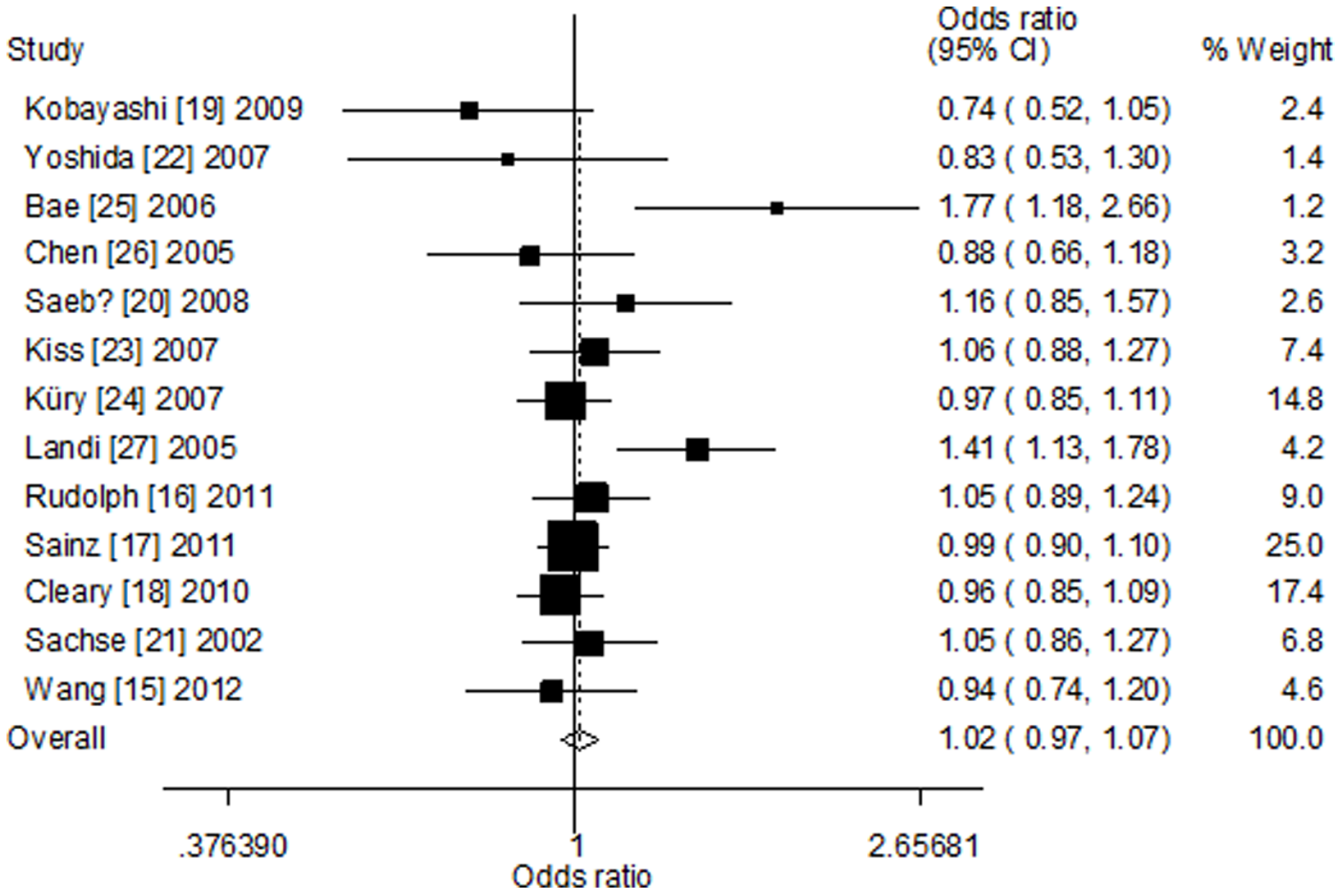
Forest plot of CYP1A2*F polymorphism and colorectal cancer risk among overall analysis (additive model).

**Figure 3:**
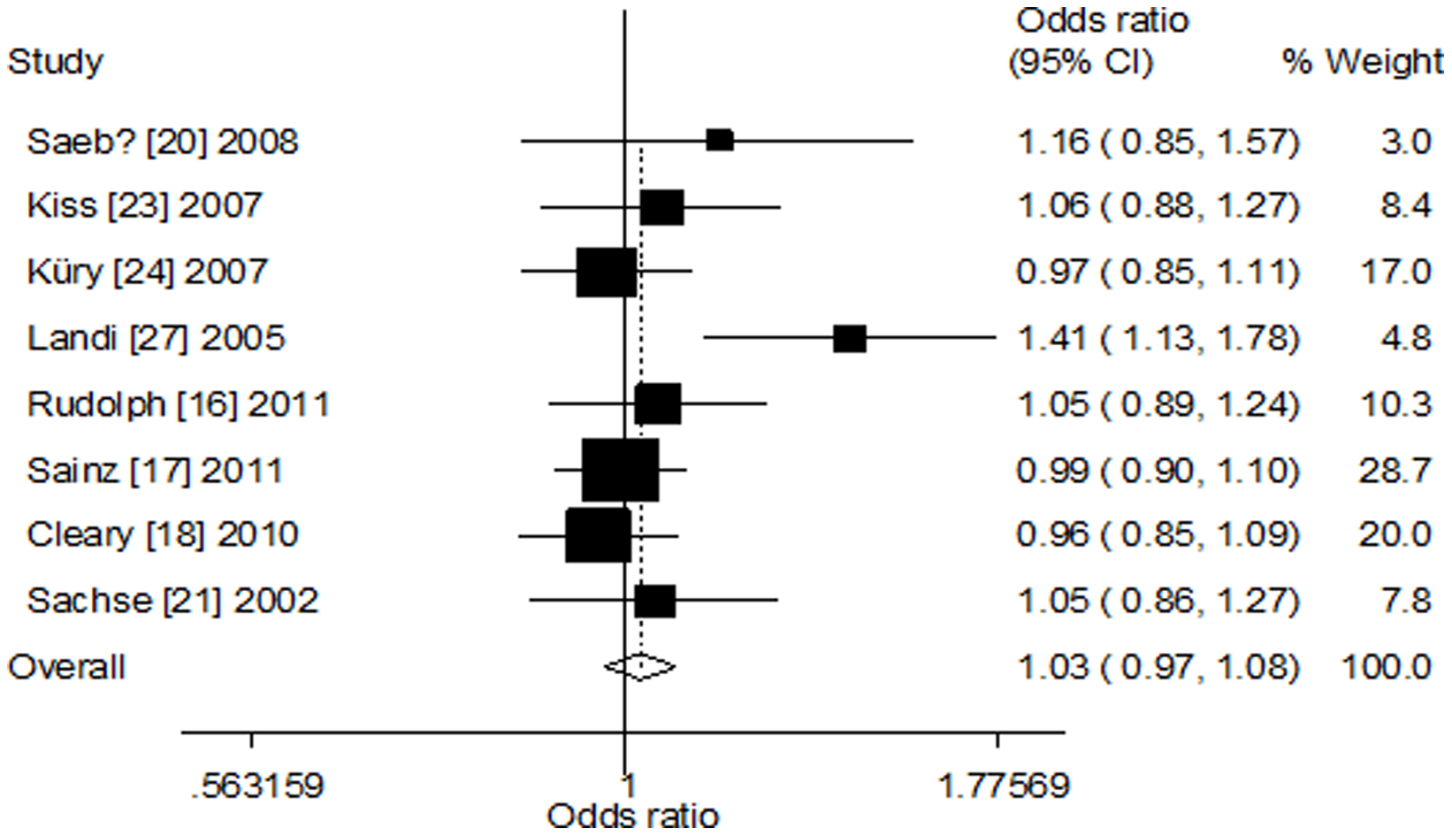
Forest plot of CYP1A2*F polymorphism and colorectal cancer risk among Caucasians (additive model).

**Figure 4:**
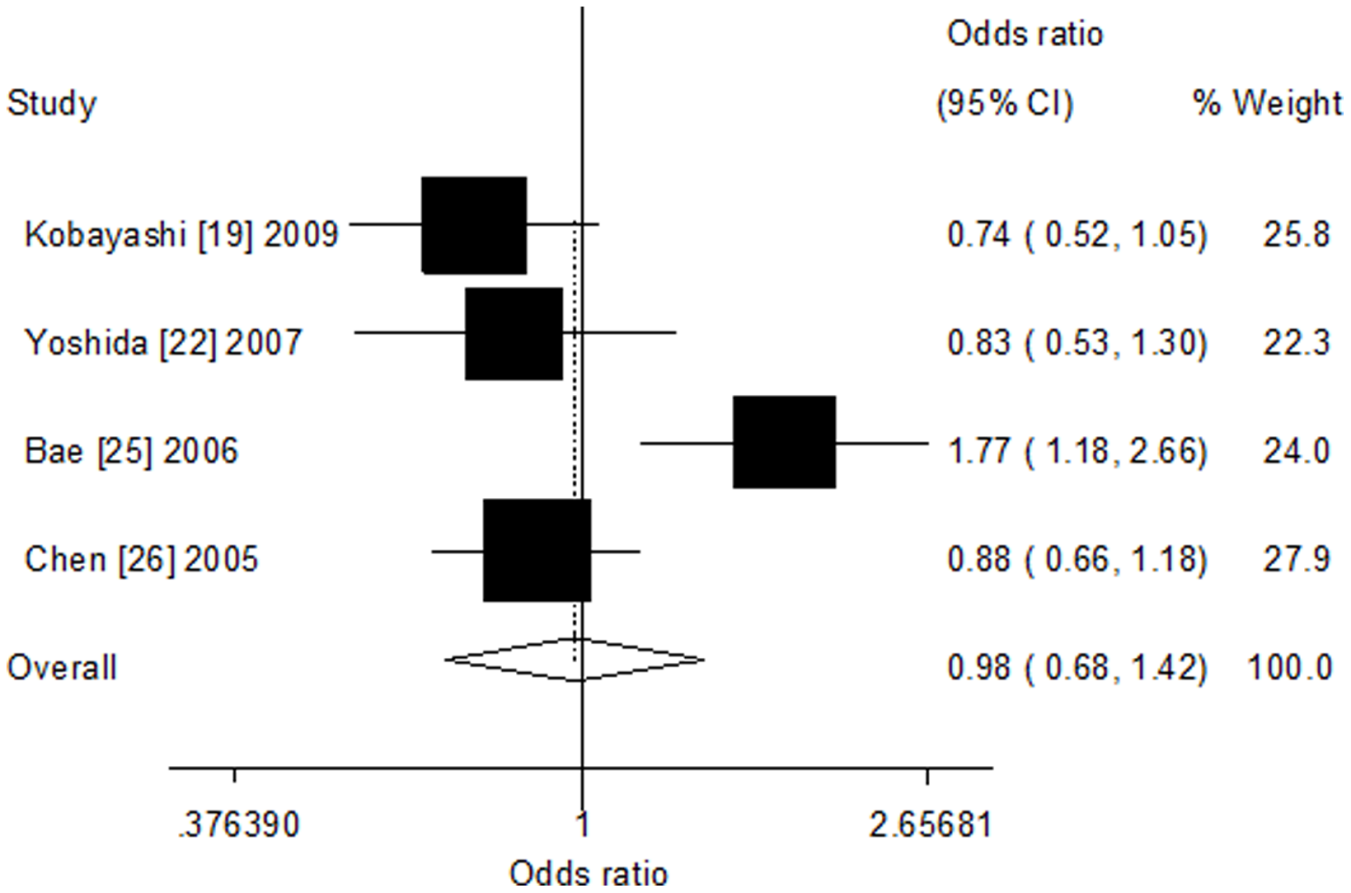
Forest plot of CYP1A2*F polymorphism and colorectal cancer risk among Asians (additive model).

**Table 2** lists the main results of the meta-analysis of CYP1A2*F polymorphism and colorectal cancer risk. Overall, no significant association was found between CYP1A2*F polymorphism and colorectal cancer risk (dominant model: OR = 1.05, 95% CI = 0.94–1.18, *P*_h_ = 0.010, *I* ^2^ = 54.1%; recessive model: OR = 1.01, 95% CI = 0.90–1.13, *P*_h_ = 0.426, *I* ^2^ = 2.0%; homozygote model: OR = 1.04, 95% CI = 0.93–1.17, *P*_h_ = 0.144, *I* ^2^ = 30.0%; heterozygote model: OR = 1.05, 95% CI = 0.94–1.17, *P*_h_ = 0.023, *I* ^2^ = 49.2%; additive model: OR = 1.03, 95% CI = 0.95–1.11, *P*_h_ = 0.026, *I* ^2^ = 48.2%, **Fig. 2**). Significant between-study heterogeneity was detected. Hence, we performed the stratified analyses according to ethnicity and source of controls. In the stratified analysis by ethnicity, no significant association was found among Caucasians (dominant model: OR = 1.02, 95% CI = 0.95–1.10, *P*_h_ = 0.233, *I* ^2^ = 24.6%; recessive model: OR = 1.06, 95% CI = 0.94–1.20, *P*_h_ = 0.387, *I* ^2^ = 5.6%; homozygote model: OR = 1.07, 95% CI = 0.94–1.21, *P*_h_ = 0.224, *I* ^2^ = 25.6%; heterozygote model: OR = 1.01, 95% CI = 0.94–1.09, *P*_h_ = 0.403, *I* ^2^ = 3.5%; additive model: OR = 1.03, 95% CI = 0.97–1.08, *P*_h_ = 0.157, *I* ^2^ = 34.0%, **Fig. 3**) and Asians (recessive model: OR = 0.78, 95% CI = 0.57–1.05, *P*_h_ = 0.681, *I* ^2^ = 0.0%; homozygote model: OR = 0.91, 95% CI = 0.49–1.68, *P*_h_ = 0.076, *I* ^2^ = 56.5%; additive model: OR = 0.98, 95% CI = 0.69–1.42, *P*_h_ = 0.009, *I* ^2^ = 74.3%, **Fig. 4**). In addition, high heterogeneity was found among Asians (dominant model: *I* ^2^ = 81.3%; heterozygote model: *I* ^2^ = 79.0). When grouped by source of control, there was still no evidence of significant association.

**Table 2:** Results of meta-analysis for CYP1A2 and CYP1B1 polymorphisms on colorectal cancer risk.^1^

Generic model		Recessive model			Dominant model			Homozygote			Heterozygote			Additive model		
CYP1A2*F	N (case/control)	OR (95%CI)	*P_h_*	*I* 2 (%)	OR (95%CI)	*P_h_*	*I* 2 (%)	OR (95%CI)	*P_h_*	*I* 2 (%)	OR (95%CI)	*P_h_*	*I* 2 (%)	OR (95%CI)	*P_h_*	*I* 2 (%)
Overall	13 (6891/7636)	1.01 (0.90–1.13)	0.426	2.0	1.05 (0.94–1.18)*	0.010	54.1	1.09 (0.93–1.17)	0.144	30.0	1.05 (0.94–1.17)*	0.023	49.2	1.03 (0.95–1.11)*	0.026	48.2
Ethnicity																
Caucasian	8 (6169/6510)	1.06 (0.94–1.20)	0.387	5.6	1.02 (0.95–1.10)	0.233	24.6	1.07 (0.94–1.21)	0.224	25.6	1.01 (0.94–1.09)	0.403	3.5	1.03 (0.97–1.08)	0.157	34.0
Asian	4 (417/769)	0.78 (0.57–1.05)	0.681	0.0	2	0.001	81.3	0.91 (0.49–1.68)*	0.076	56.5	2	0.003	79.0	0.98 (0.69–1.42)*	0.009	74.3
Source of controls																
PB	5 (4235/4689)	0.98 (0.85–1.13)	0.329	13.3	0.99 (0.91–1.08)	0.982	0.0	1.00 (0.86–1.17)	0.566	0.0	0.99 (0.91–1.09)	0.929	0.0	0.99 (0.93–1.06)	0.795	0.0
HB	7 (2351/2590)	1.06 (0.88–1.28)	0.303	16.6	1.18 (0.91–1.53)*	0.001	74.5	1.14 (0.82–1.59)*	0.040	54.5	1.20 (0.93–1.55)*	0.002	71.1	1.09 (0.92–1.30)*	0.004	69.1

Leu432Val	N (case/control)	Recessive model			Dominant model			Homozygote			Heterozygote			Additive model		
		OR (95%CI)	*P_h_*	*I* 2 (%)	OR (95%CI)	*P_h_*	*I* 2 (%)	OR (95%CI)	*P_h_*	*I* 2 (%)	OR (95%CI)	*P_h_*	*I* 2 (%)	OR (95%CI)	*P_h_*	*I* 2 (%)
Overall	12 (9219/10406)	1.05 (0.98–1.13)	0.251	20.3	1.00 (0.94–1.06)	0.770	0.0	1.04 (0.96–1.13)	0.383	6.3	0.98 (0.91–1.04)	0.687	0.0	1.02 (0.98–1.06)	0.498	0.0

Asn453Ser	N (case/control)	Recessive model			Dominant model			Homozygote			Heterozygote			Additive model		
		OR (95%CI)	*P_h_*	*I* 2 (%)	OR (95%CI)	*P_h_*	*I* 2 (%)	OR (95%CI)	*P_h_*	*I* 2 (%)	OR (95%CI)	*P_h_*	*I* 2 (%)	OR (95%CI)	*P_h_*	*I* 2 (%)
Overall	8 (6840/7761)	0.92 (0.76–1.11)	0.617	0.0	0.97 (0.87–1.08)*	0.053	49.6	0.92 (0.76–1.11)	0.685	0.0	0.97 (0.86–1.11)*	0.016	61.8	0.97 (0.91–1.03)	0.135	38.6
Source of controls																
PB	4 (3402/3943)	0.81 (0.62–1.05)	0.988	0.0	1.01 (0.86–1.19)*	0.054	60.8	0.82 (0.63–1.07)	0.996	0.0	0.90 (0.81–1.01)	0.275	22.5	0.94 (0.86–1.03)	0.183	41.1
HB	4 (3438/3818)	1.07 (0.81–1.40)	0.332	9.2	0.92 (0.83–1.02)	0.300	18.1	1.04 (0.79–1.37)	0.310	14.6	1.04 (0.87–1.23)*	0.036	64.9	1.00 (0.92–1.09)	0.143	44.8

Arg48Gly	N (case/control)	Recessive model			Dominant model			Homozygote			Heterozygote			Additive model		
		OR (95%CI)	*P_h_*	*I* 2 (%)	OR (95%CI)	*P_h_*	*I* 2 (%)	OR (95%CI)	*P_h_*	*I* 2 (%)	OR (95%CI)	*P_h_*	*I* 2 (%)	OR (95%CI)	*P_h_*	*I* 2 (%)
Overall	6 (4302/4791)	1.00 (0.86–1.16)	0.138	40.1	0.99 (0.91–1.08)	0.780	0.0	1.00 (0.86–1.16)	0.124	42.1	0.99 (0.91–1.08)	0.989	0.0	1.00 (0.93–1.06)	0.286	19.6

1 All summary ORs were calculated using fixed-effects models. In the case of significant heterogeneity (indicated by *), ORs were calculated using random-effects models.

2 The results were excluded due to high heterogeneity.

**Table 2** also lists the main results of the meta-analysis of CYP1B1 Leu432Val polymorphism and colorectal cancer risk. Overall, no significant association was found between CYP1B1 Leu432Val polymorphism and colorectal cancer susceptibility (dominant model: OR = 1.00, 95% CI = 0.94–1.06, *P*_h_ = 0.770, *I* ^2^ = 0.0%; recessive model: OR = 1.05, 95% CI = 0.98–1.13, *P*_h_ = 0.251, *I* ^2^ = 20.3%; homozygote model: OR = 1.04, 95% CI = 0.96–1.13, *P*_h_ = 0.383, *I* ^2^ = 6.3%; heterozygote model: OR = 0.98, 95% CI = 0.91–1.04, *P*_h_ = 0.687, *I* ^2^ = 0.0%; additive model: OR = 1.02, 95% CI = 0.98–1.06, *P*_h_ = 0.498, *I* ^2^ = 0.0%).

**Table 2** also lists the main results of the meta-analysis of CYP1B1 Asn453Ser polymorphism and colorectal cancer risk. Overall, no significant association was found between CYP1B1 Asn453Ser polymorphism and colorectal cancer susceptibility (dominant model: OR = 0.97, 95% CI = 0.87–1.08, *P*_h_ = 0.053, *I* ^2^ = 49.6%; recessive model: OR = 0.92, 95% CI = 0.76–1.11, *P*_h_ = 0.617, *I* ^2^ = 0.0%; homozygote model: OR = 0.92, 95% CI = 0.76–1.11, *P*_h_ = 0.685, *I* ^2^ = 0.0%; heterozygote model: OR = 0.97, 95% CI = 0.86–1.11, *P*_h_ = 0.016, *I* ^2^ = 61.8%; additive model: OR = 0.97, 95% CI = 0.91–1.03, *P*_h_ = 0.135, *I* ^2^ = 38.6%). Significant between-study heterogeneity was detected. Hence, we performed the stratified analysis according to source of controls. And in the subgroup analysis by source of controls, there was still no significant association detected in any genetic model.

**Table 2** also lists the main results of the meta-analysis of CYP1B1 Arg48Gly polymorphism and colorectal cancer risk. Overall, no significant association was found between CYP1B1 Arg48Gly polymorphism and colorectal cancer susceptibility (dominant model: OR = 0.99, 95% CI = 0.91–1.08, *P*_h_ = 0.780, *I* ^2^ = 0.0%; recessive model: OR = 1.00, 95% CI = 0.86–1.16, *P*_h_ = 0.138, *I* ^2^ = 40.1%; homozygote model: OR = 1.00, 95% CI = 0.86–1.16, *P*_h_ = 0.124, *I* ^2^ = 42.1%; heterozygote model: OR = 0.99, 95% CI = 0.91–1.08, *P*_h_ = 0.989, *I* ^2^ = 0.0%; additive model: OR = 0.97, 95% CI = 0.91–1.03, *P*_h_ = 0.135, *I* ^2^ = 38.6%).

### Test of heterogeneity and sensitivity

There was significant heterogeneity among these studies for dominant model comparison (*P*_h_ = 0.008 for CYP1A2*F and *P*_h_ = 0.053 for CYP1B1 Asn453Ser), heterozygote model comparison (*P*_h_ = 0.020 for CYP1A2*F and *P*_h_ = 0.016 for CYP1B1 Asn453Ser) and additive model comparison (*P*_h_ = 0.022 for CYP1A2*F). Then, we assessed the source of heterogeneity by ethnicity and source of controls. We found that ethnicity and source of controls (*data not shown*) did not contribute to substantial heterogeneity. Sensitivity analysis was conducted to determine whether modification of the inclusion criteria of this meta-analysis affected the results. Although the sample size for cases and controls in all eligible studies ranged from 175 to 2,455, the corresponding pooled ORs were not qualitatively altered with or without the study of small sample. In addition, a single study involved in the meta–analysis was deleted each time to reflect the influence of individual data set to the pooled ORs. The results were also not qualitatively altered.

### Publication bias

**Figure 5:**
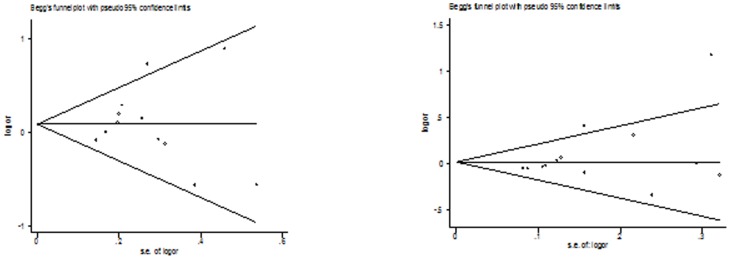
Begg's funnel plot of the meta-analysis of colorectal cancer risk and CYP1A2*F polymorphism (homozygote model and dominant model).

Both Begg's funnel plot and Egger's test were performed to assess the publication bias of literatures. The Egger's test results and Begg's funnel plot (**Fig. 5** **,** **6**) suggested no evidence of publication bias in the meta-analysis of CYP1A2*F (*P* = 0.160 for dominant model, *P* = 0.714 for recessive model, *P* = 0.862 for homozygote model; *P* = 0.248 for heterozygote model; *P* = 0.462 for additive model) and Leu432Val (*P* = 0.749 for dominant model, *P* = 0.864 for recessive model, *P* = 0.991 for homozygote model; *P* = 0.721 for heterozygote model; *P* = 0.689 for additive model), although possible publication bias was suggested for Asn453Ser polymorphism with colorectal cancer risk in additive model and recessive model and for Arg48Gly with colorectal cancer risk in any genetic model. This might be a limitation for meta-analysis of Arg48Gly and Asn453Ser polymorphisms, especially those with small sample size, are less likely to be published. **Figure 7** **,** **8** lists the Duval and Tweedie nonparametric “trim and fill” methods funnel plot in additive model and recessive model. Adjusting for possible publication bias using the Duval and Tweedie nonparametric “trim and fill” method for overall studies, the results did not change between Arg48Gly and Asn453Ser polymorphism with colorectal cancer risk.

**Figure 6:**
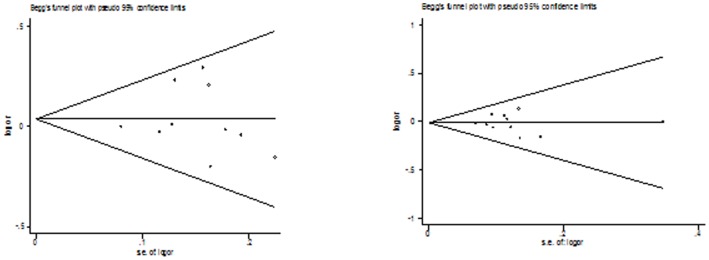
Begg's funnel plot of the meta-analysis of colorectal cancer risk and CYP1B1 Leu432Val polymorphism (homozygote model and dominant model).

**Figure 7:**
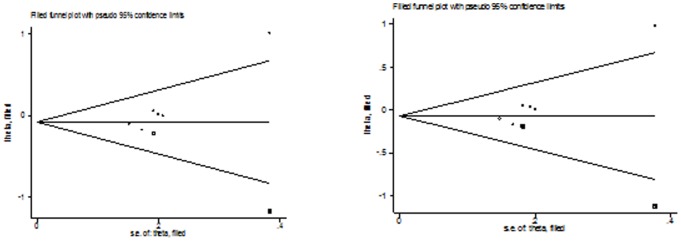
The Duval and Tweedie nonparametric “trim and fill” method's funnel plot funnel plot of the meta-analysis of colorectal cancer risk and CYP1B1 Arg48Gly polymorphism (additive model and dominant model).

**Figure 8:**
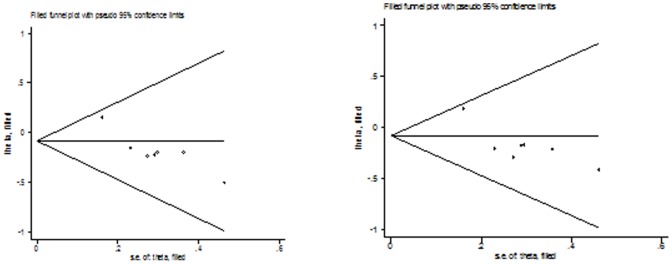
The Duval and Tweedie nonparametric “trim and fill” method's funnel plot funnel plot of the meta-analysis of colorectal cancer risk and CYP1B1 Asn453Ser polymorphism (additive model and dominant model).

## Discussion

CYP1B1 is commonly over-expressed inhumanmalignancies and activates a variety of carcinogens. For example, CYP1B1 catalyzes both the formation of dihydrodiols of specific PAHs and their subsequent oxidation to carcinogenic dihydrodiol epoxides. The importance of CYP1B1 in chemical carcinogens is well illustrated in animal models in which metabolites of CYP1B1 were shown to induce Prostate cancer risk [54], [55]. CYP 1A2 is an important gene in catalyzing 2- and 4-hydroxylations of estrogens and metabolism of carcinogens. A major reason for the limited number of studies of heterocyclic amine (HCA) and cancer risk is the difficulty of assessing human exposure to HCAs. HCA concentrations depend on cooking methods and the “doneness” level of the meat or fish, hampering the development of a complete and standardized database of concentrations; any estimation of dietary intake from food-frequency questionnaires (FFQs) is thus likely to result in misclassification. Like other environmental chemical carcinogens, HCAs require metabolic activation by host enzymes to become genotoxic. Phase I enzymes, including cytochrome P450 1A2, can metabolically activate carcinogens to form genotoxic electrophilic intermediates [56]. The relative activity of these metabolizing enzymes, which is in large part genetically determined, is thought to be an important host determinant of cancer incidence. A number of epidemiological studies have evaluated the association between CYP1A2*F, CYP1B1 Leu432Val, Asn453Ser, and Arg48Gly polymorphisms and colorectal cancer risk, but the results remain inconclusive. In order to resolve this conflict, this meta-analysis of 39 eligible studies including 5,817 cases and 6,544 controls for CYP1A2*F (from 13 studies), 9,219 cases and 10,406 controls for CYP1B1 Leu432Val (from 12 studies), 6,840 cases and 7,761 controls for CYP1B1 Asn453Ser (from 8 studies), and 4,302 cases and 4,791 controls for CYP1B1 Arg48Gly (from 6 studies) was performed to derive a more precise estimation of the association between CYP1A2*F, CYP1B1 Leu432Val, Asn453Ser, and Arg48Gly polymorphisms and risk of colorectal cancer.

Overall, no significant association was found between CYP1A2*F, CYP1B1 Leu432Val, Asn453Ser, and Arg48Gly when all the eligible studies were pooled into the meta-analysis. And in the subgroup, no evidence of significant association was also observed in any subgroup. Sachse et al. [33] in 2002 and Küry et al. [24] in 2007 reported that CYP1B1 Leu432Val was not associated with increased the risk of colorectal cancer. Landi et al. [27] and Huber et al. [37] in 2005 reported that CYP1B1 Leu432Val and Asn453Ser polymorphisms were also not associated with increased the risk of colorectal cancer. Cleary et al. [18] in 2010 found that CYP1B1 Leu432Val, Asn453Ser, and Arg48Gly were not associated with increased the risk of colorectal cancer. Sachse et al. [21] in 2002, Yoshida et al. [22] in 2007, Kiss et al. [23] in 2007, and Cleary et al. [18] reported that CYP1A2*F, was not associated with increased the risk of colorectal cancer. The results of our meta-analysis supported the negative association between CYP1A2*F, CYP1B1 Leu432Val, Asn453Ser, and Arg48Gly polymorphisms and colorectal cancer risk. However, a careful matching should be considered in future larger genetic association studies including multiple ethnic groups.

We noticed that 3 previous meta-analysis [33], [57], [58] had been reported on the colorectal cancer risk with CYP1A2*F, CYP1B1 Leu432Val, and Asn453Ser polymorphisms. We have read with great interest the meta-analysis by Mei et al. [57] and Xie et al. [58]. Mei et al. [35] had 7 studies including 6,375 cases and 7,003 controls. The pooled analysis suggested that no significant association was found between the CYP1B1 Asn453Ser polymorphism and the risk of colorectal cancer among Caucasians. Xie et al. [58] had 10 studies including 8,466 cases and 9,301 for Leu432Val. Their meta-analyses suggested that CYP1B1 Leu432Val were not associated with colorectal cancer risk. However, the study of Northwood et al. [30] should be excluded in the meta-analyses of Mei et al. [57] and Xie et al. [58] because they performed CYP1B1 Leu432Val with colorectal adenoma risk but not colorectal cancer. Adopting the same search strategy as Mei et al. [57] and Xie et al. [58], we identified 4 additional eligible studies, which have not been included in the meta-analysis of Xie et al. [36]. Worthy of note, these 4 studies included 3,638 samples. Zhao et al. [33] included 11 studies. Their meta-analysis suggests that the CYP1A2*F polymorphism is a protective factor against CRC among Asians. The OR (95% CI) reported by Zhao et al. [33] for the study by Bae et al. [25] do not seem in line with the OR (95% CI) provided by Bae et al. [25] in their original publication. The OR (95% CI) reported by Zhao et al. [33] in additive model are 0.56 (0.38–0.84). Interestingly enough, after carefully studying the OR (95% CI) presented by Bae et al. [25], The OR (95% CI) were 1.77 (1.18–2.66). In addition, the study of Wang et al. [59] should be excluded in the meta-analysis of Zhao et al. [33] because the data on CYP1A2*F polymorphism with colorectal cancer risk did not be found in the study of Wang et al. [59]. Adopting the same search strategy as Zhao et al. [33], we identified 3 additional eligible studies, which have not been included in the meta-analysis of Zhao et al. [33]. Worthy of note, these 3 studies included 2687 samples. Having analyzed an almost twofold larger number of studies than the previous meta-analysis [33], [57], [58], our results seem to confirm and establish the trend in the meta-analysis of CYP1A2*F, CYP1B1 Leu432Val, Asn453Ser, and Arg48Gly polymorphisms that the data by the previous meta-analysis [33], [57], [58] had indicated. The results of the present meta-analysis are not in accordance with those reported by Zhao et al. [33]. Our meta-analysis indicates that CYP1A2*F are not associated with colorectal cancer risk.

There are several limitations in this meta-analysis. First, the controls were not uniformly defined. Although most of them were common populations, some controls were population-based; other controls were hospital-based. Hence, non–differential misclassification bias is possible. Second, in the subgroup analysis may have had insufficient statistical power to check an association, Third, we were also unable to examine the interactions among gene-environment, lacking of the original data of the included studies limited our further evaluation of potential interactions, which may be an important component of the association between CYP1A2*F, CYP1B1 Leu432Val, Asn453Ser, and Arg48Gly polymorphisms and environment and colorectal cancer risk. Last, our results were based on unadjusted published estimates. Because of data limitations, we were unable to adjust them such as age and alcohol consumption et al.

In summary, this meta-analysis indicates that CYP1A2*F, CYP1B1 Leu432Val, Asn453Ser, and Arg48Gly are not associated with colorectal cancer. However, it is necessary to conduct large sample studies using standardized unbiased genotyping methods, homogeneous cancer patients and well-matched controls. Moreover, further studies estimating the effect of gene–gene and gene–environment interactions may eventually lead to our better, comprehensive understanding of the association between the CYP1A2*F, CYP1B1 Leu432Val, Asn453Ser, and Arg48Gly polymorphisms and colorectal cancer risk.

## Supporting Information

Checklist S1 **PRISMA Checklist.**
(DOC)

## References

[1] Ishibe N, Sinha R, Hein DW, Kulldorff M, Strickland P, et al (2002) Genetic polymorphisms in heterocyclic amine metabolism and risk of colorectal adenomas. Pharmacogenetics 12: 145–150. 11875368 10.1097/00008571-200203000-00008

[2] Jemal A, Siegel R, Xu J, Ward E (2010) Cancer statistics, 2010. CA Cancer J Clin 60: 277–300. 20610543 10.3322/caac.20073

[3] Gerhardsson de Verdier M, Hagman U, Steineck G, Rieger A, Norell SE (1990) Diet, body mass and colorectal cancer: a case-referent study in Stockholm. Int J Cancer 46: 832–838. 2172171 10.1002/ijc.2910460514

[4] Cope GF, Wyatt JI, Pinder IF, Lee PN, Heatley RV, et al (1991) Alcohol consumption in patients with colorectal adenomatous polyps. Gut 32: 70–72. 1991640 10.1136/gut.32.1.70 PMC1379217

[5] Levin B (1992) Nutrition and colorectal cancer. Cancer 70: 1723–1726. 1516026 10.1002/1097-0142(19920915)70:4+<1723::aid-cncr2820701612>3.0.co;2-3

[6] Ishibe N, Sinha R, Hein DW, Kulldorff M, Strickland P, et al (2001) Physical activity in relation to cancer of the colon and rectum in a cohort of male smokers. Cancer Epidemiol Biomarkers Prev 10: 265–268. 11303597

[7] Kushi L, Giovannucci E (2002) Dietary fat and cancer. Am J Med 113: 63S–70S. 12566141 10.1016/s0002-9343(01)00994-9

[8] Potter JD (1996) Nutrition and colorectal cancer. Cancer Causes Control 7: 127–146. 8850441 10.1007/BF00115644

[9] Norat T, Bingham S, Ferrari P, Slimani N, Jenab M, et al (2005) Meat, fish, and colorectal cancer risk: the European Prospective Investigation into cancer and nutrition. J Natl Cancer Indy 97: 906–916. 10.1093/jnci/dji164 PMC1913932 15956652

[10] Buters JT, Sakai S, Richter T, Pineau T, Alexander DL, et al (1999) Cytochrome P450 CYP1B1 determines susceptibility to 7, 12-dimethylbenz (a) anthraceneinduced lymphomas. Proc Natl Acad Sci 96: 1977–1982. 10051580 10.1073/pnas.96.5.1977 PMC26722

[11] Shimada T, Hayes CL, Yamazaki H, Amin S, Hecht SS, et al (1996) Activation of chemically diverse procarcinogens by human cytochrome P-450 1B1. Cancer Res 56: 2979–2984. 8674051

[12] Murray GI, Taylor MC, McFadyen MC, McKay JA, Greenlee WF, et al (1997) Tumor-specific expression of cytochrome P450 CYP1B1. Cancer Res 57: 3026–3031. 9230218

[13] Kim JH, Stansbury KH, Walker NJ, Trush MA, Strickland PT, et al (1998) Metabolism of benzo[a]pyrene and benzo[a]pyrene-7,8-diol by human cytochrome P450 1B1. Carcinogenesis 19: 1847–1853. 9806168 10.1093/carcin/19.10.1847

[14] Stoilov I, Akarsu AN, Alozie I, Child A, Barsoum-Homsy M, et al (1998) Sequence analysis and homology modeling suggest that primary congenital glaucoma on 2p21 results from mutations disrupting either the hinge region or the conserved core structures of Cytochrome P4501B1. Am J Hum Genet 62: 573–584. 9497261 10.1086/301764 PMC1376958

[15] Wang J, Joshi AD, Corral R, Siegmund KD, Marchand LL, et al (2012) Carcinogen metabolism genes, red meat and poultry intake, and colorectal cancer risk. Int J Cancer 130: 1898–1907. 21618522 10.1002/ijc.26199 PMC3883510

[16] Rudolph A, Sainz J, Hein R, Hoffmeister M, Frank B, et al (2011) Modification of menopausal hormone therapy-associated colorectal cancer risk by polymorphisms in sex steroid signaling, metabolism and transport related genes. Endocr Relat Cancer 18: 371–384. 21490239 10.1530/ERC-11-0057

[17] Sainz J, Rudolph A, Hein R, Hoffmeister M, Buch S, et al (2011) Association of genetic polymorphisms in ESR2, HSD17B1, ABCB1, and SHBG genes with colorectal cancer risk. Endocr Relat Cancer 18: 265–276. 21317201 10.1530/ERC-10-0264

[18] Cleary SP, Cotterchio M, Shi E, Gallinger S, Harper P (2010) Cigarette smoking, genetic variants in carcinogen-metabolizing enzymes, and colorectal cancer risk. Am J Epidemiol 172: 1000–1014. 20937634 10.1093/aje/kwq245 PMC2984254

[19] Kobayashi M, Otani T, Iwasaki M, Natsukawa S, Shaura K, et al (2009) Association between dietary heterocyclic amine levels, genetic polymorphisms of NAT2, CYP1A1, and CYP1A2 and risk of colorectal cancer: a hospital-based case-control study in Japan. Scand J Gastroenterol 44: 952–959. 19452301 10.1080/00365520902964721

[20] Saebø M, Skjelbred CF, Brekke Li K, Bowitz Lothe IM, Hagen PC, et al (2008) CYP1A2 164 A->C polymorphism, cigarette smoking, consumption of well-done red meat and risk of developing colorectal adenomas and carcinomas. Anticancer Res 28: 2289–2295. 18751408

[21] Sachse C, Smith G, Wilkie MJ, Barrett JH, Waxman R, et al (2002) A pharmacogenetic study to investigate the role of dietary carcinogens in the etiology of colorectal cancer. Carcinogenesis 23: 1839–1849. 12419832 10.1093/carcin/23.11.1839

[22] Yoshida K, Osawa K, Kasahara M, Miyaishi A, Nakanishi K, et al (2007) Association of CYP1A1, CYP1A2, GSTM1 and NAT2 gene polymorphisms with colorectal cancer and smoking. Asian Pac J Cancer Prev 8: 438–444. 18159984

[23] Kiss I, Orsós Z, Gombos K, Bogner B, Csejtei A, et al (2007) Association between allelic polymorphisms of metabolizing enzymes (CYP 1A1, CYP 1A2, CYP 2E1, mEH) and occurrence of colorectal cancer in Hungary. Anticancer Res 27: 2931–2937. 17695473

[24] Küry S, Buecher B, Robiou-du-Pont S, Scoul C, Sébille V, et al (2007) Combinations of cytochrome P450 gene polymorphisms enhancing the risk for sporadic colorectal cancer related to red meat consumption. Cancer Epidemiol Biomarkers Prev 16: 1460–1467. 17627011 10.1158/1055-9965.EPI-07-0236

[25] Bae SY, Choi SK, Kim KR, Park CS, Lee SK, et al (2006) Effects of genetic polymorphisms of MDR1, FMO3 and CYP1A2 on susceptibility to colorectal cancer in Koreans. Cancer Sci 97: 774–779. 16800822 10.1111/j.1349-7006.2006.00241.x PMC11160064

[26] Chen K, Jin MJ, Fan CH, Song L, Jiang QT, et al (2005) A case–control study on the association between genetic polymorphisms of metabolic enzymes and the risk of colorectal cancer. Zhonghua Liu Xing Bing Xue Za Zhi 26: 659–664. 16471212

[27] Landi S, Gemignani F, Moreno V, Gioia-Patricola L, Chabrier A, et al (2005) A comprehensive analysis of phase I and phase II metabolism gene polymorphisms and risk of colorectal cancer. Pharmacogenet Genomics 15: 535–546. 16006997 10.1097/01.fpc.0000165904.48994.3d

[28] Rudolph A, Sainz J, Hein R, Hoffmeister M, Frank B, et al (2011) Modification of menopausal hormone therapy-associated colorectal cancer risk by polymorphisms in sex steroid signaling, metabolism and transport related genes. Endocr Relat Cancer 18: 371–384. 21490239 10.1530/ERC-11-0057

[29] Sainz J, Rudolph A, Hein R, Hoffmeister M, Buch S, et al (2011) Association of genetic polymorphisms in ESR2, HSD17B1, ABCB1, and SHBG genes with colorectal cancer risk. Endocr Relat Cancer 18: 265–276. 21317201 10.1530/ERC-10-0264

[30] Northwood EL, Elliott F, Forman D, Barrett JH, Wilkie MJ, et al (2010) Polymorphisms in xenobiotic metabolizing enzymes and diet influence colorectal adenoma risk. Pharmacogenet Genomics 20: 315–326. 20375710 10.1097/FPC.0b013e3283395c6a

[31] Hlavata I, Vrana D, Smerhovsky Z, Pardini B, Naccarati A, et al (2010) Association between exposure-relevant polymorphisms in CYP1B1, EPHX1, NQO1, GSTM1, GSTP1 and GSTT1 and risk of colorectal cancer in a Czech population. Oncol Rep 24: 1347–1353. 20878130 10.3892/or_00000992

[32] Trubicka J, Grabowska-Kłujszo E, Suchy J, Masojć B, Serrano-Fernandez P, et al (2010) Variant alleles of the CYP1B1 gene are associated with colorectal cancer susceptibility. BMC Cancer 10: 420. 20701755 10.1186/1471-2407-10-420 PMC2929240

[33] Zhao Y, Chen ZX, Rewuti A, Ma YS, Wang XF, et al (2013) Quantitative Assessment of the Influence of Cytochrome P450 1A2 Gene Polymorphism and Colorectal Cancer Risk. PLoS One 8: e71481. 23951174 10.1371/journal.pone.0071481 PMC3741149

[34] Cotterchio M, Boucher BA, Manno M, Gallinger S, Okey AB, et al (2008) Red meat intake, doneness, polymorphisms in genes that encode carcinogen-metabolizing enzymes, and colorectal cancer risk. Cancer Epidemiol Biomarkers Prev 17: 3098–3107. 18990750 10.1158/1055-9965.EPI-08-0341 PMC2751598

[35] Küry S, Buecher B, Robiou-du-Pont S, Scoul C, Sébille V, et al (2007) Combinations of cytochrome P450 gene polymorphisms enhancing the risk for sporadic colorectal cancer related to red meat consumption. Cancer Epidemiol Biomarkers Prev 16: 1460–1467. 17627011 10.1158/1055-9965.EPI-07-0236

[36] Bethke L, Webb E, Sellick G, Rudd M, Penegar S, et al (2007) Polymorphisms in the cytochrome P450 genes CYP1A2, CYP1B1, CYP3A4, CYP3A5, CYP11A1, CYP17A1, CYP19A1 and colorectal cancer risk. BMC Cancer 7: 123. 17615053 10.1186/1471-2407-7-123 PMC1925111

[37] Huber A, Bentz EK, Schneeberger C, Huber JC, Hefler L, et al (2005) Ten polymorphisms of estrogen-metabolizing genes and a family history of colon cancer-an association study of multiple gene-gene interactions. J Soc Gynecol Investig 12: e51–e54. 10.1016/j.jsgi.2005.07.003 16202920

[38] Landi S, Gemignani F, Moreno V, Gioia-Patricola L, Chabrier A, et al (2005) A comprehensive analysis of phase I and phase II metabolism gene polymorphisms and risk of colorectal cancer. Pharmacogenet Genomics 15: 535–546. 16006997 10.1097/01.fpc.0000165904.48994.3d

[39] Wang J, Joshi AD, Corral R, Siegmund KD, Marchand LL, et al (2012) Carcinogen metabolism genes, red meat and poultry intake, and colorectal cancer risk. Int J Cancer 130: 1898–1907. 21618522 10.1002/ijc.26199 PMC3883510

[40] Yamazaki H, Shaw PM, Guengerich FP, Shimada T (1998) Role of cytochromes P450 1A2 and 3A4 in the oxidation of estradiol and estrone in human liver microsomes. Chem Res Toxicol 11: 659–665. 9625734 10.1021/tx970217f

[41] Nebert DW, Dalton TP (2006) The role of cytochrome P450 enzymes in endogenous signalling pathways and environmental carcinogenesis. Nat Rev Cancer 6: 947–960. 17128211 10.1038/nrc2015

[42] Tsuchiya Y, Nakajima M, Yokoi T (2005) Cytochrome P450-mediated metabolism of estrogens and its regulation in human. Cancer Lett 227: 115–124. 16112414 10.1016/j.canlet.2004.10.007

[43] Nebert DW, Dalton TP, Okey AB, Gonzalez FJ (2004) Role of aryl hydrocarbon receptor-mediated induction of the CYP1 enzymes in environmental toxicity and cancer. J Biol Chem 279: 23847–23850. 15028720 10.1074/jbc.R400004200

[44] Nebert DW, McKinnon RA, Puga A (1996) Human drugmetabolizing enzyme polymorphisms: effects on risk of toxicity and cancer. DNA Cell Biol 15: 273–280. 8639263 10.1089/dna.1996.15.273

[45] Eaton DL, Gallagher EP, Bammler TK, Kunze KL (1995) Role of cytochrome P4501A2 in chemical carcinogenesis: implications for human variability in expression and enzyme activity. Pharmacogenetics 5: 259–27420. 8563766 10.1097/00008571-199510000-00001

[46] Nakajima M, Yokoi T, Mizutani M, Kinoshita M, Funayama M, et al (1999) Genetic polymorphism in the 5′-flanking region of humanCYP1A2 gene: effect on theCYP1A2 inducibility in humans,. J Biochem 125: 803–808. 10101295 10.1093/oxfordjournals.jbchem.a022352

[47] Davey SG, Egger M (1997) Meta-analyses of randomized controlled trials. Lancet 350: 1182. 9343537 10.1016/s0140-6736(05)63833-0

[48] Ioannidis JP, Boffetta P, Little J, O'Brien TR, Uitterlinden AG, et al (2008) Assessment of cumulative evidence on genetic associations: interim guidelines. Int J Epidemiol 37: 120–132. 17898028 10.1093/ije/dym159

[49] Mantel N, Haenszel W (1959) Statistical aspects of the analysis of data from retrospective studies of disease. Natl Cancer Inst 22: 719–778. 13655060

[50] DerSimonian R, Laird N (1986) Meta-analysis in clinical trials. Control Clin Trials 7: 177–188. 3802833 10.1016/0197-2456(86)90046-2

[51] Klug SJ, Ressing M, Koenig J, Abba MC, Agorastos T, et al (2009) TP53 codon 72 polymorphism and cervical cancer: a pooled analysis of individual data from 49 studies. Lancet Oncol 10: 772–784. 19625214 10.1016/S1470-2045(09)70187-1

[52] Begg CB, Mazumdar M (1994) Operating characteristics of a rank correlation test for publication bias. Biometrics 50: 1088–1101. 7786990

[53] Egger M, Smith DG, Schneider M, Minder C (1997) Bias in meta-analysis detected by a simple, graphical test. BMJ 315: 629–634. 9310563 10.1136/bmj.315.7109.629 PMC2127453

[54] Williams JA, Martin FL, Muir GH, Hewer A, Grover PL, et al (2000) Metabolic activation of carcinogens and expression of various cytochromes P450 in human prostate tissue. Carcinogenesis 21: 1683–1689. 10964100 10.1093/carcin/21.9.1683

[55] Cavalieri EL, Devanesan P, Bosland MC, Badawi AF, Rogan EG (2002) Catechol estrogen metabolites and conjugates in different regions of the prostate of Noble rats treated with 4-hydroxyestradiol: implications for estrogen-induced initiation of prostate cancer. Carcinogenesis 23: 329–333. 11872641 10.1093/carcin/23.2.329

[56] McManus ME, Burgess WM, Veronese ME, Huggett A, Quattrochi LC, et al (1990) Metabolism of 2-acetylaminofl uorene and benzo(a)pyrene and activation of food-derived heterocyclic amine mutagens by human cytochromes P-450. Cancer Res 50: 3367–3376. 2334931

[57] Mei Q, Zhou D, Han J, Lu H, Tang B (2012) CYP1B1 Asn453Ser polymorphism and colorectal cancer risk: a meta-analysis. Metabolism. 2012 61: 1321–1329. 10.1016/j.metabol.2012.02.010 22459615

[58] Xie Y, Liu GQ, Miao XY, Liu Y, Zhou W, et al (2012) CYP1B1 Leu432Val polymorphism and colorectal cancer risk among Caucasians: a meta-analysis. Tumour Biol 33: 809–816. 22190224 10.1007/s13277-011-0298-7

[59] Wang H, Yamamoto JF, Caberto C, Saltzman B, Decker R, et al (2010) Genetic variation in the bioactivation pathway for polycyclic hydrocarbons and heterocyclic amines in relation to risk of colorectal neoplasia. Carcinogenesis 32: 203–209. 21081473 10.1093/carcin/bgq237 PMC3026844

